# Morpheme Matching Based Text Tokenization for a Scarce Resourced Language

**DOI:** 10.1371/journal.pone.0068178

**Published:** 2013-08-21

**Authors:** Zobia Rehman, Waqas Anwar, Usama Ijaz Bajwa, Wang Xuan, Zhou Chaoying

**Affiliations:** 1 Department of Computer Science, COMSATS Institute of Information Technology, Abbottabad, Pakistan; 2 Harbin Institute of Technology, Shenzhen, Graduate School, China; UC Davis School of Medicine, United States of America

## Abstract

Text tokenization is a fundamental pre-processing step for almost all the information processing applications. This task is nontrivial for the scarce resourced languages such as Urdu, as there is inconsistent use of space between words. In this paper a morpheme matching based approach has been proposed for Urdu text tokenization, along with some other algorithms to solve the additional issues of boundary detection of compound words, affixation, reduplication, names and abbreviations. This study resulted into 97.28% precision, 93.71% recall, and 95.46% F1-measure; while tokenizing a corpus of 57000 words by using a morpheme list with 6400 entries.

## Introduction

Urdu is a morphologically rich language, spoken by more than 150 million people of the world; either as their mother tongue or second language. It is composed of many different languages of the world, e.g., Arabic, Persian, Turkish, Hindi, Sanskrit, and English. It frequently adopts new words from the other languages as well. It is a bidirectional language and uses Arabic based orthography, whereas its morphology is influenced by all the above mentioned languages [1].

Tokenization is a very first step for numerous language processing tasks, e.g., part of speech tagging, machine translation, spell checking, sentence boundary detection, information retrieval, and information extraction. It is simpler for inflectional languages such as English, where space is used as word delimiter. In some of the Asian languages, space is frequently used even after each character, e.g., Chinese, Thai, and Lao. In such languages, the challenge for tokenization is to omit the space which comes between the characters forming a single word. In hand written Urdu text there is no convention of delimiters; words are written in continuation without any space between them. There are two types of characters in Urdu; joiners and non joiners as shown in table 1 and 2 respectively. Joiners are the characters which can occupy the initial, medial or final forms in the word. If a word ends with a joiner character and no delimiter is used after it then it will join itself with its following word, resulting into a vague one, which will not be understandable even for the native speaker of the language. That's why space is used after such words just to make them reader understandable. Sometimes instead of this space a special Urdu character, Zero Width Non Joiner (ZWNJ) is used to keep such words apart from their followings.

**Table 1:** Non-Joiner Urdu Alphabets.

ا د ڈ ذ ر ز ڑ ژ و ے

**Table 2:** Joiner Urdu Alphabets.

ب پ ت ٹ ث ج چ ح خ س ش ص ض ط ظ ع غ ف ق ک گ ل م ن ہ ء ھ ی

Non joiners are the characters which do not concatenate themselves with their following characters or words; therefore it is not needed to place any delimiter after a word ending at a non joiner.

The uneven use of delimiters makes the tokenization of Urdu text more difficult. During tokenization it is also needed to assign single boundary to compound words, words with affixations, reduplicated words, names, and abbreviations.

Tokenization approach proposed in this paper is based on morpheme matching. Forward maximum matching, dynamic maximum matching and dynamic maximum matching along with maximum likelihood approach have been used to split the Urdu text into tokens. Some other algorithms also have been designed to solve the issues of compound words, affixation, reduplication, names and abbreviations. This work has been tested over a corpus of 57000 words using a lexicon with 6400 entries. It produced 97.28% precision, 93.71% recall, and 95.46% F1-measure with all known words in the corpus.

### 1. Issues of Urdu text tokenization

It is easy to tokenize the string by just splitting it using the space between words. But it is difficult for the languages which do not use space or use it inconsistently between words. Space is not used in hand written Urdu text and it is the one's own job to identify the individual words in continuum string. In computerized Urdu text documents, space is used occasionally according to diverse nature of Urdu characters. The problems of Urdu text tokenization can be divided into two; space inclusion issues and space exclusion issues.

#### 1.1. Space inclusion issues

In computerized Urdu text, it is needed to insert space between words or add ZWNJ at the end of first word, if it ends at a joiner character.

In table 3, (I) string is written without inter word space and (II) with space at the end of each word. It is obvious that all the words end at non joiners, that's why in example (I) and (II), both give the same meanings. Native speaker can understand that both of the examples have same words but example (I) will appear as a single vague word for the machine.

**Table 3:** Words ending at non joiners.

اسدشہرسےباہرجاپہنچا (I)	اسد شہر سے باہر جا پہنچا (II)
Asad reached out of the city.	

ZWNJ is used between two words, if it is needed to keep them apart from each other. But it does not help to identify a word boundary; rather it helps to look them apart from each other. For example, in the table 4, there is a string “پرانیسڑک” (old track), in this the two words are separated by an additional ZWNJ character.

**Table 4:** ZWNJ between words.

(old track) پرانیسڑک
(Words without space or ZWNJ)
(old track) پرانی سڑک
(Words separated by space)
(old track) پرانیسڑک
(Words separated by ZWNJ)

#### 1.2. Space exclusion issues

Space exclusion is another issue of text tokenization. Sometimes it is needed to insert space between the words which collectively give the single meaning. In tokenization process, these words should be assigned a single boundary, while ignoring the space between them.

Table 5 shows some examples of space exclusion issue.

**Table 5:** Space exclusion issues.

Word	Category of the word
روٹی کپڑا (basic needs of life)	Compound
نظم و ضبط (discipline)	
حد نظر (scene limit)	
دن بدن (day by day)	Reduplication
صبح صبح (early morning)	
ٹھيک ٹھاک (absolutely fine)	
بيش قيمت (expensive)	Prefixation
ان تھک (hard work)	
آلہ کار (apparatus)	Suffixation
دہشت یگرد (terrorism)	
جنوبی افريقہ (South Africa)	Proper Noun with more than one word
زينب نور (Zainab Noor)	
ايش ٹرے (ash tray)	English words
نيٹ ورک (network)	
ايم قريشی (M. Qureshi)	Abbreviations
اين ايل پی (NLP)	

### 2. Tokenization techniques

There are numerous tokenization techniques available for the various languages of the world, e.g., rule based techniques [2] [3], statistical techniques [4], fuzzy techniques [5], lexical techniques [6], [7], and feature based techniques [8]. Significant work has also been done for Arabic [9] and Persian language [10] [11], which are closer to Urdu because of the same script.

In [12] Thai language text has been segmented using the longest matching technique. Algorithm reads the input text from left to right and searches for the longest match in the dictionary. If a match is found but it does not allow the algorithm to find rest of the words in the dictionary, then algorithm will back track and will search for another suitable match. This work produced 97.03% accuracy for Thai language text (composed of all known words).

In [13] the authors used forward maximum matching to segment Chinese text and they reported an error rate of 0.26% for 1.2 million characters. In this work a lexicon of 85855 words has been used and words in it have been divided according to their length. For efficient searching, authors placed all single length words in one table and all the words with length 2 in a separate table. They divided the words with length 3 into prefixes of length 2 and suffixes of length 1, while the words with four characters have been divided into prefixes and suffixes of length 2. Words with length greater than four have been divided into prefixes, infixes and suffixes of length 2. In all combinations each prefix was pointing to the corresponding suffix.

In [4] Chinese text has been statistically segmented using mutual information value. Mutual information of the characters was computed and statistical methods were applied to segment the text. They divided input text into consecutive sequences of characters. For every character in a phrase the bi-gram mutual information value was computed. Characters with the highest bi-gram mutual information value were considered the words and removed from the consecutive sequences of characters. The process was repeated until the last phrase consisted of words of length 1 or 2. This approach produced 73.49% precision and 73.90% recall for the articles obtained from the 442 Chinese news papers.

In [14] Thai text has been segmented using Ripper. It is an algorithm that learns the prepositional rules and constructs a rule set. This rule set is used to classify the training data. In this approach, these rules have been applied on the N-best segmentations, which were obtained after applying maximum matching technique along with POS tagger on the input text. This technique produced 91.27% and 89% precision for a test corpus of 2500 sentences, using context independent and context dependent features respectively.

In [9] rule based approach has been implemented to tokenize the Arabic script. In the very first step authors delimited main tokens on the basis of white spaces. In next step three different models have been designed to detect sub tokens, clitics, and stems inside the main tokens. The first model used Arabic morphological analyzer to identify the sub tokens; while the second model identified clitics with the help of clitic guesser and clitic transducer. The final model was also a morphological analyzer to identify the token boundary between clitic and stem. In the next step multiword expressions were delimited in the tokens, white spaces were normalized and tokenization ambiguities were removed.

Authors in [11] developed a tokenizer for Persian language by combining dictionary based and rule based approaches. This tokenizer delimits words, multipart verbs, abbreviations, numbers, dates and proper nouns.

In [15] authors developed a segmenter for Urdu language using the bilingual corpora and statistical techniques. The task of space omission in Urdu text has been completed in two main phases; in first phase the merged words have been delimited and in the second phase the individual words identified inside the merged words. This segmenter has been tested for 1.61 million words and it showed 99.15% accuracy for the words facing the space omission problem. The study in [16] used n-gram technique along with maximum matching to build a segmenter for Urdu language and achieved 95.8% accuracy.

## Proposed Methods to Tokenize Urdu Text

In proposed work, Urdu text has been tokenized by using forward maximum matching algorithm, dynamic maximum matching algorithm, and the combination of dynamic maximum matching along with maximum likelihood approach. In preprocessing phase of our approach, we removed the diacritics, ZWNJ, and white spaces from the text. So the text could acquire the form of space free string, which could be further divided into morphemes by using available algorithms and morpheme look-up list. Once the basic morphemes were available from the input text, we applied our supporting algorithms to join them where needed.

### 1. Forward maximum matching

In forward maximum matching, string tokenization is started from right to left. Urdu character string without any space or ZWNJ and list of free morphemes (sorted and reversed) have been passed to the algorithm and the algorithm returned the list of individual tokens of the string.

**1.1. Algorithm**

1. Search in the morpheme list for the free morpheme that matches with the start of the string.
2. If it is found, append it to the token list and strip it out from the string but if no match is found, strip a single character and append it with the token list.
3. Repeat the above two steps until the string gets empty.
4. Finally search for all the single characters in the token list and concatenate them to the previous token in the list.

The above algorithm is explained in the following example and its output as shown in the table 6 and 7.

**Table 6:** Output of forward maximum matching.

سعودی (Saudi)

**Table 7:** Output of forward maximum matching.

سعودی (Saudi)	عرب (Arab)

“**سعودی عرب**” (Saudi Arab) is an Urdu text string, having free morphemes [“**سعود**” (Saud), “سعودی” (Saudi), “دی” (di), “دیع” (diA), “عر” (Ar), “**عرب**” (Arab), “رب” (Rab)]. All these morphemes are obtained from the list of free morphemes. For the tokenization of the Urdu text it is required to insert and delete the space from the text according to conditions. To resolve this ambiguity, algorithm removes all the spaces and ZWNJ characters from the input text and tokenizes it according to the list of free morphemes. Tokens demanding no space between them are merged to form a single token by applying some other supporting algorithms. These algorithms are discussed in coming sections. After removing space, the string acquires the form “**سعودیعرب**” (Saudi Arab). Algorithm sorts and reverses the morpheme list and new morpheme list becomes [“**عرب**” (Arab), “عر” (Ar), “سعودی” (Saudi), “سعود” (Saud), “دیع” (diA), “دی” (di), “رب” (rab)]. Algorithm searches from left to right, in the morpheme list for the morpheme which matches with the start of the string. It finds “سعودی” (Saudi) in the list and strips it from the string and string becomes “**عرب**” (Arab). This morpheme is striped out from the string and stored in the token list.

Algorithm starts its search again in the morpheme list for the remaining characters of the string. Searching from left to right it finds “**عرب**” (Arab) in the very start, as the string starts with this morpheme so it is stripped out from the string and appended to the token list.

### 2. Dynamic maximum matching

Forward maximum matching gives only one tokenization sequence; while dynamic matching gives all the possible tokenization sequences of the given string according to the available morpheme list. If it can not find any match then it splits the string into characters. Total number of single characters in each tokenization sequence is considered as number of errors in it. It selects one having minimum number of tokens, as best tokenization sequence. But if there are more than one tokenization sequences with same number of words, it selects one of them having minimum number of errors.

**2.1. Algorithm**

1. In the list of free morphemes find all those morphemes which match with the start of the string.
2. Once they are found, populate the 2-D array with them which is used to store the all possible combinations of input string, number of tokens and number of errors in each combination.
3. If no match is found then strip a single character from the input string and store it in the 2D-array and update the error field against that specific segmentation sequence.
4. At the end select one with the minimum number of tokens and errors.
5. Concatenate each single character with the previous token in the list.

Consider the same example which has been used in forward maximum matching algorithm; for the above algorithm, it is explained in the tables 8, 9, 10, 11. String to be tokenized is “**سعودی عرب**” (Saudi Arab) along with the list of free morphemes [“**سعود**” (Saud), “**سعودی**” (Saudi), “**دی**” (di), “**دیع**” (diA), “**عر**” (Ar), “**عرب**” (Arab), “**رب**” (rab)]. Like forward maximum matching, it will eliminate white spaces, diacritics and ZWNJ characters from input string. So the input string will look like “**سعودیعرب**” (Saudi Arab). It will create a 2-D matrix for different segmentation sequences. The second last column in the 2-D matrix is used to represent number of tokens in the row, whereas the last column is used to represent the number of errors in it.

**Table 8:** Output of dynamic maximum matching.

سعود (Saud)	1	0
سعودی (Saudi)	1	0

**Table 9:** Output of dynamic maximum matching.

سعود (Saud)	ی(i)	2	1
سعودی (Saudi)		1	0

**Table 10:** Output of dynamic maximum matching.

سعود(Saud)	ی(i)	عر(Ar)	3	1
سعودی(Saudi)			1	0
سعود(Saud)	ی(i)	عرب(arab)	3	1

**Table 11:** Output of dynamic maximum matching.

سعود(Saud)	ی(i)	عر(Ar)	ب(ab)	4	2
سعودی(Saudi)	عر(Ar)	ب(ab)		3	1
سعود(Saud)	ی(i)	عرب(Arab)		3	1
سعودی(Saudi)	عرب(Arab)			2	0

In the first step, the algorithm will search in the list of free morphemes for all the possible morphemes which match with the start of the input string. It will find “**سعود**” (Saud) and “**سعودی**” (Saudi), and will store them in the 2-D matrix as shown in table 8.

In next step it will take the token “**سعود**” (Saud) and will find morphemes in the list that follow this token in the input string. As it does not find any match, so it will read only next character from the input string and will store it in the array after incrementing the error variable by 1.

For the first array, it will start its search again for the morphemes following “**ی**” (i) in the string. As it finds two morphemes, therefore it will store a copy of this row in the next empty row available, to append the corresponding morphemes.

Entire process will continue until all possible segmentation sequences are completed for the input string. The final 2-D matrix will be as shown in table 11.

To find the best segmentation amongst all, the algorithm will compare the number of tokens and the number of errors in all the segmentation sequences. One with the minimum number of tokens will be considered the best segmentation sequence for the input string. If more than one segmentation sequences have the same number of tokens then the one having minimum number of errors will be selected. In the example given in table 11, last segmentation with two tokens and without any error will be selected.

### 3. Dynamic maximum matching along with maximum likelihood approach

This technique works on more than one possible outcomes of the dynamic matching algorithm. It calculates probability of each token in the corpus and computes cumulative probability of each tokenization sequence. Tokenization sequence with highest cumulative probability is considered the most optimal tokenization scheme for the input string.

If the DMM algorithm returns more than one token combinations with equal number of tokens and errors, then bigram probability of each token will be calculated for each combination and the model will return the one with highest cumulative probability value P(T) = ∩_i = 1−n_ P(t_i_|t_i−1_) (Eq. 1) [17]. In Eq. 1, T represents the contestant combination having all possible tokens and t represents the individual tokens in T.

Consider the example given in table 12.

**Table 12:** Segmentations produced by dynamic matching.

اس	نے	کہا	کہ	اسے	جنے	دو	Correct
اس	نے	کہا	کہا	سے	جنے	دو	Incorrect
He said let him in. (Correct).							

Suppose these two segmentations are obtained from dynamic matching, both having equal number of words and no error. In order to select one of them, both of these will be passed to the bi-gram statistical model. Cumulative probability values 1.6e-11 and 2.4e-16 have been calculated the segmentations in the first and second row of the table respectively. As the first segmentation has the highest value of cumulative probability, therefore it will be selected as the best tokenization sequence.

### 4. Supporting algorithms

Forward maximum matching and dynamic maximum matching techniques tokenize the input text into free morphemes, but to handle the issues of affixation, compound words, names, and abbreviations following algorithms have been designed.

- Algorithm for compound word generation
- Algorithm for prefixation
- Algorithm for suffixation
- Algorithm for full reduplication
- Algorithm for partial reduplication
- Algorithm to handle names and abbreviations

**4.1. Algorithm for compound word generation**

1. In the token list, group two consecutive tokens such that neither should be ‘اور’ (and) nor ‘و’ (and).
2. Find a match for the new token in the list of compound morphemes. If a match is found then replace the first token in the token list with new token and remove the next token from the list.
3. If the second token is ‘اور’ (and) or ‘و’ (and) then group three consecutive tokens to create the new one. Find a match for it in the compound word list. If it is found then replace the first token in the list with this and remove next two tokens from the token list. Group the tokens in a way such that if the preceding token ends with joiner character then embed the ZWNJ between preceding and following token.

The words shown in table 13 and 14 are the lists of tokens generated by any of the forward maximum matching or dynamic maximum matching algorithm. If table 13 is passed to compound word generation algorithm, it will search for the every element of the token list, in the list of compound words. For the given example, if it reads the token “**محنت**” (hard work) and finds it also in the compound words list; in this case it will read previous token of it “**بہت**” (very) and the next token “**و**” (and). According to algorithm first condition is not met as previous token “**بہت**” (very) is not in the compound words list, therefore for the second condition algorithm will read the token “**مشقت**” (struggle) as next token. Now “**محنت**” (hard work), “**و**” (and) and “**مشقت**” (struggle) will become previous, current and next tokens respectively. The first condition of algorithm is not satisfied as the current token is “**و**” (and), therefore it will check for the second condition. As previous, current and next, all the tokens are available in the list of compound morphemes and current token is “**و**” (and), so it will join them while embedding ZWNJ after previous token to form the compound word “**محنتومشقت**” (hard work). It will replace previous token “**محنت**” (hard work) with this compound word while removing “**و**” (and) and “**مشقت**” (struggle) from the list of tokens.

**Table 13:** Compound word generation.

وہ	بہت	محنت	و	مشقت	سے	کام	کرتا	تھا
He had been working very hard.						

**Table 14:** Compound word generation.

وہ	بہت	محنتومشقت	سے	کام	کرتا	تھا
He had been working very hard.						

**4.2. Algorithm for prefixation**

1. Search for every element of the token list, in the list of prefixes.
2. If it is found then group it with the next token in the token list. If the previous token ends with a joiner character then embed ZWNJ between previous and the next token.

Consider the examples shown in table 15 and 16. The above algorithm will search for every token in the list of prefixes until it finds a match or array traversing is completed. For the given example after finding the token “**نا**” (un) in the list of prefixes, it will read the token (next token) “**اہل**” (able) which follows it in the token list and will concatenate both of them to form “**نااہل**” (unable). Further it will replace “**نا**” (un) with the new token “**نااہل**” (unable) and will remove “**اہل**” (able) from the token list. Output of this algorithm will be as given in table 16.

**Table 15:** Example of prefixation.

سب	نا	اہل	تھے	ايک	مشکل	حل	نہ	کر	سکے
They were even unable to solve a single problem.									

**Table 16:** Example of prefixation.

سب	نااہل	تھے	جو	مشکل	حل	نہ	کر	سکے
They were even unable to solve a single problem.								

**4.3. Algorithm for suffixation**

1. After fixing the prefixes reverse the order of the token list.
2. Search for every token in the list of suffixes.
3. If a match is found then concatenate first token at the end of the next, in the token list, such that if next token ends with a joiner then embed ZWNJ between two tokens.
4. Reverse the order of the token list.

The different stages of the suffixation process have been shown in the tables 17, 18, 19, 20.

**Table 17:** Example of suffixation.

اس	نے	بہت	متاثر	کن	کام	کيا
He performed impressively.						

**Table 18:** Example of suffixation.

کيا	کام	کن	متاثر	بہت	نے	اس
He performed impressively.						

**Table 19:** Example of suffixation.

کيا	کام	متاثر کن	بہت	نے	اس
He performed impressively.					

**Table 20:** Example of suffixation.

اس	نے	بہت	متاثر کن	کام	کيا
He performed impressively.					

Algorithm will reverse the list of tokens shown in table 17.

Now it will start reading the tokens from left and for each token it will try to find a match in the list of suffixes. In the given example, it finds “**کن**” in the suffix list and reads next token “**متاثر**” (impressed). Further both of these tokens will be concatenated to form “**متاثر کن** ” (impressing). Now suffix “**کن**” will be removed from the token list and “**متاثر**” (impressed) will be replaced with “**متاثر کن**” (impressing).

Finally the list will be reversed to get the real order of tokens as in the input token list.

**4.4. Algorithm for full reduplication**

1. For every token in the list of token, compare each to the next in the token list.
2. If both are equal then combine them to form a new token. If the token ends at a joiner character, then embed a ZWNJ between them.

Table 21 and 22, show the input and output for the full reduplication algorithm respectively.

**Table 21:** Example of full reduplication.

اس	نے	دو	دو	ہار	خريدے
He bought two necklaces.					

**Table 22:** Example of full reduplication.

اس	نے	دودو	ہار	خريدے
He bought two necklaces.				

Algorithm will read the tokens in the array, in the form of the pair of previous and next token. If a pair contains similar contents then it joins them to form a single token. In the given example, when algorithm reads the token “دو” (two) as previous token and next to it is also “دو” (two), therefore it will join both of these to form “دودو” (two) and will replace the previous token in the list with this newly concatenated token. This algorithm will also remove the next token “دو” (two) from the token list.

**4.5. Algorithm for partial reduplication**

1. For every token in the token list, compare the length of two consecutive tokens. If they are equal in length and the length is not less than 4 [16], then compare them character by character. If one character is dissimilar, it means they can be combined to form a partial reduplicated word.
2. If there is difference of one character in the length of two tokens and excluding the first character of the second token, both the tokens are similar then combine them to form a new token. If the first token ends with a joiner then embed a ZWNJ between them.

Table 23 and 24, show the input and output token lists for the partial reduplication algorithm respectively.

**Table 23:** Example of partial reduplication.

وہ	گاہے	بگاے	جيا	کرتا	تھا
He had been visiting time to time.					

**Table 24:** Example of partial reduplication.

وہ	گاہےبگاہے	آيا	کرتا	تھا
He had been visiting time to time.				

When the algorithm will start reading the above token list, in its first iteration, it will find the token “**وہ**” (he) as previous token and “گاہے” as the next. But both of these do not satisfy the condition of having three or more than three corresponding similar characters. In the next iteration the token “گاہے” will become previous token and “بگاے” the next. As both of these have more than three similar characters, so the algorithm will concatenate them by placing the ZWNJ between them and new token will become “گاہےبگاہے” (time to time). It will replace “گاہے” with newly concatenated token and will remove token “بگاے” from the token list.

**4.6. Algorithm for names and abbreviations**

1. Search for every token in the list of names and in the list of English characters, if match is found then check the previous token.
2. If the previous token is in the name list or ends with a name, it is an English character or ends with an English character, or it is not equal to ‘-’ but ends with a ‘-’ then combine both of the tokens. If first token ends with a joiner character then embed a ZWNJ between them.
3. If the newly formed token ends with the ‘کے’ then split it into two, by separating ‘کے’ from the token.

Different phases of this algorithm are shown in the tables 25, 26, 27, 28, 29. As algorithm starts reading the elements in the token list, it will find the very first token in the list of names. As it has no previous token therefore algorithm will read the next token “علی” in the token list. It will also be found in the list of names; therefore it will read the previous token of it and will search for it in the same list. As it is available in the name list also, therefore new token will be composed by concatenating both of these, while inserting ZWNJ between them. The new token “**اسدعلی**” will replace the previous token “**اسد**” in the list and the token “علی” will be removed from it, as shown in table 26.

**Table 25:** Example of names and abbreviate.

اسد	علی	نے	يو	۔	ايس	۔	اے	۔	جانا	ہے
Asad Ali has to visit U.S.A.										

**Table 26:** Example of names and abbreviations.

اسدعلی	نے	يو	۔	ايس	۔	اے	۔	جانا	ہے
Asad Ali has to visit U.S.A.									

**Table 27:** Example of names and abbreviations.

اسدعلی	نے	يو۔	ايس	۔	اے	۔	جانا	ہے
Asad Ali has to visit U.S.A.								

**Table 28:** Example of names and abbreviations.

اسدعلی	نے	يو۔ ايس	۔	اے	۔	جانا	ہے
Asad Ali decided to visit U.S.A.							

**Table 29:** Example of names and abbreviations.

اسدعلی	نے	يو۔ ايس۔ اے اے۔	جانا	ہے
Asad Ali has to visit U.S.A.				

Onwards it will read the next token “نے” in the token list, but as it does not exist in the list of names, neither it is a ‘۔’ nor an English character, therefore algorithm will look for the next token in the list. It reads “يو” but previous token “نے” does not satisfy the condition; therefore algorithm will go for the next token. It finds ‘۔’ as next token and “يو” as the previous token. As the condition of the algorithm is satisfied, so both of these tokens will be combined as “يو۔”. New token will replace the previous token “يو” in the token list and “۔” will be removed from the list, as shown in table 27. Algorithm reads the next token “ايس” in the token list, it is also an English character and according to the algorithm, previous token “يو۔” ends with “۔”, therefore both of these will be merged as shown in table 28. Similarly the next token “۔” will be joined to previous token “يو۔ ايس” to form “**يو۔ ايس۔**” and the same process will be followed for the next two tokens “اے” and “۔”. The output of this process is shown in the table 29. At last the algorithm will check in the list for the token which starts with a name and ends with “**کے**”. If a match is found, it will split it into name and “**کے**”; as in the table 29 there is no such case, therefore it is the final output for the given example.

## Experimental Results and Discussion

Experimental results are calculated by tokenizing the corpus with 57000 words by using a morpheme list containing 6400 free morphemes. Test corpus has been tokenized by using three different approaches; forward maximum matching, dynamic maximum matching and dynamic maximum matching along with maximum likelihood approach. Following two paragraphs illustrate how the corpus has been tokenized by using the three mentioned techniques.

Suppose there is a string “**اس نے سنا کہ اسے جانے دو**” (He heard that let him leave.) and list of free morphemes related to it [**“نے”, “ناک”, “کہا”, “کہ”, “سے”, “سنا”, “سن”, “دو”, “جانے”, “جا”, “اسے”, “اس”**]. Maximum matching will return the list of tokens [**“اس”, “نے”, “سنا”, “کہا”, “سے”, “جانے”, “دو”**] for the input string and it contains two words “**کہا**” and “**سے**” which have been tokenized incorrectly.

Dynamic matching tokenizes the similar string into [“**اس**”, “**نے**”, “**سنا**”, “**کہا**”, “**سے**”, “**جانے**”, “**دو**”] and [“**اس**”, “**نے**”, “**سنا**”, “**کہ**”, “**اسے**”, “**جانے**”, “**دو**”], with equal number of tokens and without any error. It will select the first tokenization sequence with two incorrect tokens, because both sequences appear same to it, as they have equal number of tokens and errors. But if it is combined with maximum likelihood approach then it will select the three best segmentations out of all produced tokenization schemes and will compute bi-gram probability for each. So for the given example, dynamic maximum matching along with maximum likelihood will return the tokenization sequence [“**اس**”, “**نے**”, “**سنا**”, “**کہ**”, “**اسے**”, “**جانے**”, “**دو**”], containing all correct tokens in it.

**Figure 1:**
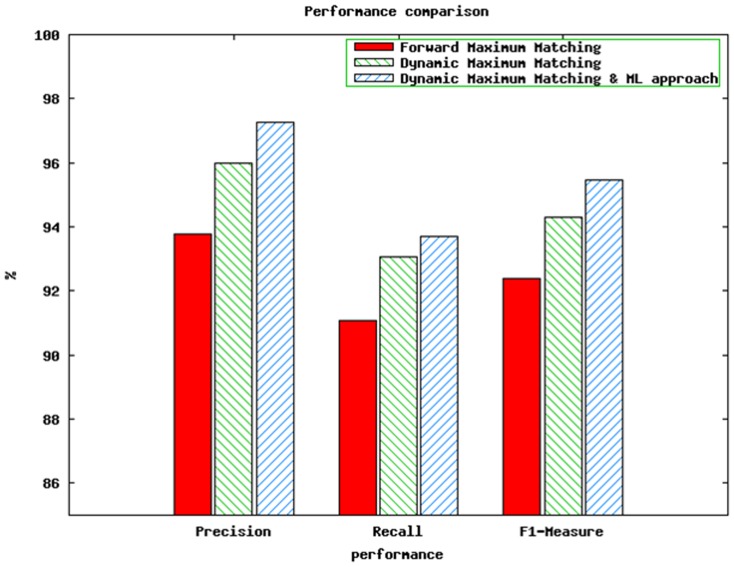
Performance comparison.

The results obtained after applying the proposed three different techniques over 57000 words are shown in figure 1. Using forward maximum matching 93.78% precision, 91.06% recall, and 92.39% F1-measure are obtained. Dynamic matching produced 96.00% precision, 93.06% recall, and 94.31% F1-measure. Best results have been seen by using dynamic maximum matching along with maximum likelihood approach, which are 97.28% precision, 93.71% recall, and 95.46% F1-measure.

The study in [15], achieved 99.29% recall and 99.38% precision for Urdu merged word recognition component. The author used Urdu, Hindi, and English morphological rules to find the merged words in the text. He used longest matching, maximum matching and statistical rules to fix only the space omission issues in Urdu text. Author had the advantage of availability of the bilingual corpus which had been helpful, while solving the ambiguities seen during maximum matching process.

In a study [16] similar to ours, the same corpus was used while applying same techniques but in a different way. Authors initially segmented the text with the available spaces between the words, further they searched for orthographic words inside the available segments for space omission problem. After fixing space omission problem they applied their rules for space insertion errors. This study reported an accuracy of 95.8%. Following formulas are used for precision, recall and F1-measure;

Precision = number of correct tokens returned by tokenizer/total number of tokens returned by tokenizer

Recall = number of correct tokens returned by tokenizer/total number of tokens in Corpus

F1-measure = 2*Precision*Recall/(Precision+Recall)

## Conclusion

The problem of tokenizing Urdu text strings revolves around the insertion and deletion of the space between the words. In the hand written Urdu text, there is no use of space between the words but in case of the computerized text files space is inserted after the words ending at joiner characters (characters which join themselves with the following characters). In this work Urdu text has been tokenized using three different approaches; forward maximum matching, dynamic maximum matching, and dynamic maximum matching along with maximum likelihood approach. All of these approaches work with some other algorithms which have been proposed to resolve the issues of identification of compound words, affixations, reduplication, names, and abbreviations. This work produced up to 97.28% precision, 93.71% recall, and 95.46% F1-measure with the test data comprising of 57000 words. The work proposed in this paper is more dependent on the corpus; it definitely affects the results, if there are unseen words (words not available in the corpus) in the text to be segmented. In future we are aimed to develop a tokenization method which would be least dependant on the corpus, and using machine learning techniques, would be able to learn the morphological patterns of the valid morphemes in Urdu text. So instead of searching for morphemes in a corpus, it could be searched for specific morphological patterns in the text in order to tokenize it.
